# Supplementary material for: Mutated clones driving leukemic transformation are already detectable at the single-cell level in CD34-positive cells in the chronic phase of primary myelofibrosis
Source: NPJ Precis Oncol. 2021 Feb 4;5:4. doi: 10.1038/s41698-021-00144-9 (PMC7862275; doi:10.1038/s41698-021-00144-9)
Supplement: Supplementary file 2 — Supplementary Material [file 41698_2021_144_MOESM2_ESM.pdf]

Supplementary information related to the manuscript:

"Mutated clones driving leukemic transformation are already detectable at the single cell level in CD34-positive cells in the chronic phase of primary myelofibrosis"

SUPPLEMENTARY DISCUSSION

ScRNA-seq clustering analysis

By means of graph based clustering task on Partek® Flow® we defined cell clusters specific for each sample; in particular this analysis identified 5 clusters in T1 and 4 clusters in T2 and T3 **(Supplementary Fig. 7).** In order to define the identity of these clusters we classified cells according to their expression profile using Blueprint ENCODE dataset in SingleR package. Furthermore, we defined clusters' marker genes by means of Partek® Flow® and we compared them with those identified by several authors in different subpopulations of hematopoietic stem/progenitor cells[1-3].

As shown in **Supplementary Fig. 7c,** T1 cluster 1 (T1.1) includes foremost hematopoietic stem cells (HSCs) and multipotent progenitors (MPPs) according to SingleR classification and is characterized by the expression of HSC markers such as ***AVP*[4], *HOPX*[5]**, ***CRHBP*[6]**, ***MEG3*[7]** and ***IGHM*[8]** (**Supplementary Data 1 and Supplementary Fig. 7d**).

MPP cells are also included in cluster 2 (T1.2) which is composed primarily by granulocyte and monocyte progenitors (GMPs) (**Supplementary Figure 7c).** This cluster is characterized by the expression of genes associated to multilineage and granulocytic progenitors such as ***SPINK2***, ***SPINS3*** and ***CLEC12A*[9]** (**Supplementary Data 1 and Supplementary Fig. 7d**).

According to SingleR classification, clusters 3 and 4 (T1.3 and T1.4) includes almost exclusively megakaryocyte-erythroid progenitors (MEPs) (**Supplementary Fig. 7c**). Marker gene analysis revealed that T1.4 cluster is characterized by the expression of ***MS4A2*[10]**, reported as a marker gene of Eosinophil Basophil and Mastocyte (Eo/B/Mast) progenitors, but also by megakaryocytic and erythroid progenitors' genes such as ***CNRIP1*[11]**, ***FCER1A*[12]** and ***HPGDS*** (**Supplementary Data 1 and Supplementary Fig. 7d**).

Conversely, in T1.3 cluster, which includes not only MEP but also Erythrocytes according to SingleR (**Supplementary Figure 7c**), cells express erythroid progenitors' specific genes such as ***ANK1*[13,14]** (**Supplementary Data 1 and Supplementary Fig. 7d**).

According to SingleR classification cluster 5 (T1.5) is composed almost exclusively by megakaryocytes (**Supplementary Figure 7c**) and is characterized by the expression of specific MK progenitor genes such as ***GP9*** and ***ITGB3*** (CD61) (**Supplementary Data 1**). Finally, another cluster was identified; it is composed by different cell types according to SingleR classification, including monocytes (data not shown). Gene expression analysis revealed the expression of marker genes identified in different lymphoid and myeloid populations according to literature findings (data not shown). We considered these 71 cells as contaminants and excluded them from analysis.

As told before, clustering analysis identified 4 cell clusters in both T2 and T3 samples and the same strategy described for Time 1 was applied in order to define cluster identity in these specimens.

As you can see in **Supplementary Fig. 7** we detected high level similarities between clusters identified in the three samples in terms of cell composition (SingleR Blueprint classifications) and marker genes expression. Indeed, T2 cluster 1 (T2.1) and T3 cluster 1 (T3.1) include HSCs, MPPs, GMPs and a small fraction of MEPs like T1 cluster 1 (T1.1) and display common marker genes such as ***AVP***, ***HOPX*** and ***CRHBP*** specific for hematopoietic stem cells as told before (**Supplementary Data 1 and Supplementary Fig. 7d,h and n**). Therefore, we classified cells belonging to these clusters as HSC_MPP.

T1 cluster 2 (T1.2) displayes a cellular composition highly similar to T2 cluster 2 (T2.2) and T3 cluster 2 (T3.2) including foremost GMPs (**Supplementary Fig. 7c, g and m).** Once again, these clusters display common marker genes associated to granulocytic or multilineage progenitors such as ***IGLL1, CLEC12A*** and ***SPNS3*** (**Supplementary Data 1 and Fig. Supplementary 7d, h and n**), therefore we decided to define these clusters as MPP_GMP.

Cells belonging to megakaryocytic or erythroid lineages (MEPs but also erythrocytes and megakaryocytes) were included in T2 and T3 clusters 3 and 4 (**Supplementary Fig. 7g and m)**. T2 cluster 3 (T2.3) and T3 cluster 4 (T3.4) are composed almost exclusively by MEPs such as T1 cluster 4 (T1.4) and shared the expression of genes reported as expressed in Eo/B/Mast progenitors but also MEPs and ERPs (i.e. ***MS4A2***, ***HPGDS*** and ***CNRIP1***) (**Supplementary Data 1 and Supplementary Fig. 7d, h and n**). This observation was consistent with recent findings demonstrating the common origin of MEPs and Eo/B/Mast progenitors[15], leading us to hypothesize that these clusters represent a more primitive state in megakaryocytic and erythroid lineage differentiation. For these reasons we grouped these clusters in MEP_1 category.

Conversely T2 cluster 4 (T2.4) and T3 cluster 3 (T3.3) include not only MEPs but also erythrocytes and megakaryocytes like T1 clusters 3 (T1.3) and 5 (T1.5) (**Supplementary Fig. 7c, g and m**), suggesting that these clusters represent late stages along this lineage. Among marker genes we identified the expression of erythroid (***ANK1*** and ***KLF1***) and megakaryocytic genes (***ITGA2B***) (**Supplementary Data 1 and Supplementary Fig. 7d, h and n).** These clusters were then included in a common MEP_2 group.

By means of AUCell task in Partek® Flow® we evaluated the activation state of gene signatures specific for hematopoietic and leukemic stem cells and progenitor cells identified by Eppert et al.[1]. As shown in **Supplementary Fig. 8** clusters belonging to HSC_MPP (T1.1, T2.1, T3.1) and MPP_GMP (T1.2, T2.2, T3.2) groups display increased activation of gene modules related to HSC phenotype (EPPERT_HSC_R and EPPERT_CE_HSC_LSC modules) (**Supplementary Fig. 8b and c**), while clusters we included in MEP_1 (T1.4, T2.3, T3.4) and MEP_2 (T1.3, T1.5, T2.4, T3.3) groups display activation of gene signatures specific for progenitor subpopulations (EPPERT_PROGENITOR) (**Supplementary Fig. 8d**). This result are therefore in agreement with our classification.

Finally, in T2 and T3 clusters 5 we identified 17 and 76 contaminating dispersed cells respectively, including myeloid and lymphoid cells (data not shown) that were removed from the analysis.

Inhibition of interferon signaling characterizes disease progression

By comparing clusters identified in the three samples we were able to identify cellular groups that share the same characteristics in T1, T2 and T3 named HSC_MPP, MPP_GMP, MEP_1 and MEP_2. Therefore, we compared gene expression between samples within the shared clusters. To better understand the molecular mechanisms responsible for changes undergoing during disease progression we studied lists of differentially expressed genes by means of IPA®.

According to IPA® analysis all cell clusters in T3 and T2 samples display a significant inactivation of interferon (IFN) signaling compared to T1 (**Supplementary Data 4 and Supplementary Fig. 9a**); this might be the effect of Ruxolitinib treatment which is able to inhibit this pathway through the downmodulation of JAK/STAT signaling. In all cell clusters belonging to T3, IFN pathway inactivation is enhanced by the upregulation of SOCS1 (**Supplementary Fig. 9a, b**), a known inhibitor of JAK/STAT signaling pathway. Furthermore, several Interferon Regulatory Factors (IRFs) were identified as inhibited upstream regulators by IPA® analysis in all clusters (**Supplementary Data 5**) due to the

downregulation of IFN-induced genes such as: STAT1, IFITM1, MX1, OAS1, IFI6 and IFIT3 (**Supplementary Fig. 9b**). Among them, IRF1, IRF3 and IRF7, are key mediators of IFN-induced immune surveillance, involved in type I HLA molecules expression. As long as the disease progresses and acquires a more aggressive phenotype, malignant cells achieve the capability to resist against immune surveillance. Surprisingly, Ruxolitinib actively contributes to this process since it is able to reduce leukemic cells' sensitivity to NK cells[16,17]. Moreover, it was demonstrated that the inhibition of IFN-γ signaling causes the downregulation of type I and type II HLA and B2M[18]. This is consistent with the enhanced immune escaping of malignant cells that lose the ability to present antigens to cytotoxic T cells and therefore can't be eradicated[19]. Our data confirm the deregulation of this axis, which is altered in other stem cells from hematological malignancies such as chronic myeloid leukemia[20].
Moreover, the downregulation of IFN signaling may also be responsible for the less differentiated state of cells in T3 and T2 samples as demonstrated by trajectory analysis (**Fig. 3g**). IFN-signaling was found to be more downregulated in MEP clusters compared to HSC_MPP and MPP_GMP (**Supplementary Data 4**). It has been described that IFN-γ is able to induce myeloid differentiation[21], and its administration has already been proposed as a therapeutic strategy in AML in order to escape the differentiative block[22]. Our results suggest that IFN-γ signaling was strong during the PMF phase, accounting for the accumulation of differentiated myeloid cells. This signal is progressively lost during disease progression, partially due to Ruxolitinib effect, therefore allowing the generation of a less differentiated blast cell population. Again, IPA® analysis supported this hypothesis since "Maturation of blood cells" category was identified among the most significantly inhibited disease and functions in T3 MEP_2 cluster (**Supplementary Data 6**).

Leukemic transformation is associated to reduced apoptosis and increased quiescence of HSPCs

According to gene enrichment analysis several disease and functions categories related to "Cell death and survival" were predicted inactivated in all the comparisons. In particular, "Cell Death of hematopoietic cells" and "Cell Death of hematopoietic progenitor cells" turned out as two of the most inhibited functions in MPP_GMP cluster in T3 vs T1 (**Supplementary Data 6**). In this comparison, IRF1 is predicted by IPA® as inactivated upstream regulator due to the downregulation of several direct targets, including the proapoptotic gene BAK1 **(Supplementary Fig. 9c and Supplementary Data 5).** Bak1 protein increases apoptosis of mouse bone marrow-derived neutrophils in cell culture that is increased by Fasl protein[23].

We can therefore hypothesize that IFN-signaling inhibition we observed in our patient during the leukemic transformation, may favor immune escape mechanisms but can also inhibit apoptosis through the regulation of ***BAK1***.

Moreover, we observed the activation of genes related to cell quiescence, a process involved in the resistance to therapy of the neoplastic clone. In particular, it has been recently demonstrated in a mouse model that Ruxolitinib treatment is more effective in proliferating cell populations rather than quiescent ones[24]. Our data shows that FOXO1 and FOXO3 are predicted by IPA® analysis as upstream regulators activated in T3 HSC_MPP and MPP_GMP cells (**Supplementary Data 5**) thus demonstrating that quiescence is activated also in leukemic stem cells. FOXO3 is also active in T3 MEP_1 cluster (**Supplementary Data 5).** These transcription factors have already been described as able to promote the maintenance of hematopoietic and leukemic stem cells[25]. FOXO transcriptional factors expression may also be related to ***TP53*** mutation. It has in fact been described that TP53-mutant is unable to promote FOXO1 and FOXO3 proteasome-mediated degradation in glioblastoma, therefore favoring the stemness of cancer cells[26].

Our results demonstrated the upregulation of several targets of the FOXO family proteins, such as ***CDKN1A*** (p21), ***PIK3IP1***, ***EGR1***, ***KLF4*** and ***KLF7***. Moreover, we observed in T3 vs T1 an increased expression of other transcriptional factors such as ***EGR1***[27], ***EGR3***[28], ***NR4A2***[29], ***MAFf***[30] and ***HES1***[31] (**Supplementary Data 3**), which co-operate to the quiescent state of HSCs and precocious myeloid-biased progenitor cells. In particular, EGR1 is shown also as active upstream regulator in all cell clusters (T3 vs. T1) and stimulates stem cell quiescence by transactivating ***AREG***, ***SOCS1*** and ***CDKN1A*** (p21) expression (**Supplementary Data 5).**

Extramedullary hematopoiesis (EMH) increases during disease progression

IPA® analysis shows that extramedullary hematopoiesis (EMH), which establishes an alternative niche for HSCs because of the fibrotic state of bone marrow (BM)[32], increases during the progression of the disease. In this niche, HSCs are protected from chemical treatments and maintain a quiescent state in order to preserve the malignant population[33]. The activation of this mechanism is confirmed by the clinical features of the patient, who has a marked splenomegaly which has only been partially reduced by Ruxolitinib treatment.

The establishment of a new niche is facilitated by stem cells’ mobilizing molecules produced by the BM, the expression of pro-invasive factors by HSCs and the presence of chemoattractant molecules in the microenvironment[34]. Our data demonstrates that the

chemokine receptor ***CXCR4*** is significantly upregulated in T2 and T3 vs T1, especially in HSC_MPP and MPP_GMP clusters (**Supplementary Data 3 and Supplementary Fig. 11**). In T3, CXCR4 upregulation extends to the more mature clusters. This modulation is a sign for disease progression, since CXCR4 is downregulated in PMF circulating CD34+ cells in comparison with healthy donors[35], but its expression is upregulated in AML being indicated as a prognostic marker for a shorter relapse-free survival and overall survival[36].

Several targets involved in cell migration have been described as induced by CXCR4 signaling and are upregulated in our dataset. Among them CXCR4 itself, the pro-leukemogenic factors ***CD69***, ***ID1*** and the pro-EMH factors ***EGR1*** and ***IL8*** can be found (**Supplementary Data 3**).

The invasion of ectopic sites by HSCs is promoted by several enzymes, such as metalloproteases, which enable extracellular matrix disruption[37]. ***MMP7*** is strongly upregulated in our data (HSC_MPP and MPP_GMP clusters in T3 vs. T1 comparison) (**Supplementary Data 3 and Supplementary Fig. 11**). One of the sources for MMP7 production is ***PTGS2*** (COX2)[38], an enzyme that is highly expressed in T3 HSPCs compared to T1. In particular, the production of prostaglandin E2 (PGE2) mediated by PTSG2 stimulates its receptors. In our dataset, ***PTGS2*** is strongly upregulated in all T3 vs T1 clusters, while in T2 vs T1 its upregulation is detected only in MEP_1 cluster **(Supplementary Data 3 and Supplementary Fig. 11**). Moreover, PGE2 and its receptors PTGER2 and 4 are active upstream regulators, according to IPA predictions, in MPP_GMP and MEP clusters of both T2 and T3 vs. T1 (**Supplementary Data 5).** PTGER2 and 4 promote through cAMP signaling pathway the transcription of several pro-EMH factors upregulated in our dataset such as ***CXCR4***[39], ***IL8***[40] and ***EGR1***[41]. Of note, upregulation of ***PTGS2*** (COX2) may also enhance immune escape since it is able to impair NK and cytotoxic T cells function[42].

Together with the upregulation of ***MMP7***, the infiltrative process is facilitated by the downregulation of ***CDH1*** in HSC_MPP and MEP_2 clusters (T3 vs. T1) and the increased expression of ***RHOB*** in HSC_MPP and MPP_GMP clusters of both T2 and T3 vs T1 (**Supplementary Data 3**). This protein in fact facilitates membrane blebbing and blebby ameboid migration of leukemic cells[43].

As previously said, EMH requires the cooperation between cells and microenvironment. Besides IL8, other chemoattractant cytokines have been identified by IPA analysis as activated upstream regulators in our dataset. Indeed, PDFGB and PF4 turned out among the strongest activated upstream regulators in all cell clusters in T3 sample (**Supplementary**

Data 5). Among the pro-migratory targets induced by PDGFB we found ***AREG, PTGS2, SGK1, SERPINE1, IER2*** and ***RHOB***.

Leukemic transformation is associated to reduced differentiation

IPA® analysis identified "Maturation of blood cells" among the most significantly inhibited disease and functions in T3 MEP_2 cluster (**Supplementary Data 6).** This is consistent with the less primed state of leukemic hematopoietic progenitors. In this category we found upregulated genes favoring the differentiative block, such as ***CD69*** and ***KLF2*** (**Supplementary Data 3**). On the other hand, we identified several downregulated genes which stimulate hematopoietic differentiation, such as ***CDH1***, ***DDX58*** and ***HOXA3*** (**Supplementary Data 3).**

Moreover, the downregulation of IFN signaling may contribute to immune escape but may also be responsible for the less differentiated state of these cells. Indeed, as described in the main text, IFN-signaling inhibition was more evident in MEP clusters compared to the stem ones. IFN-γ has been described as able to induce myeloid differentiation and its administration has already been proposed as a therapeutic strategy in AML in order to escape the differentiative block[22].

SUPPLEMENTARY FIGURES

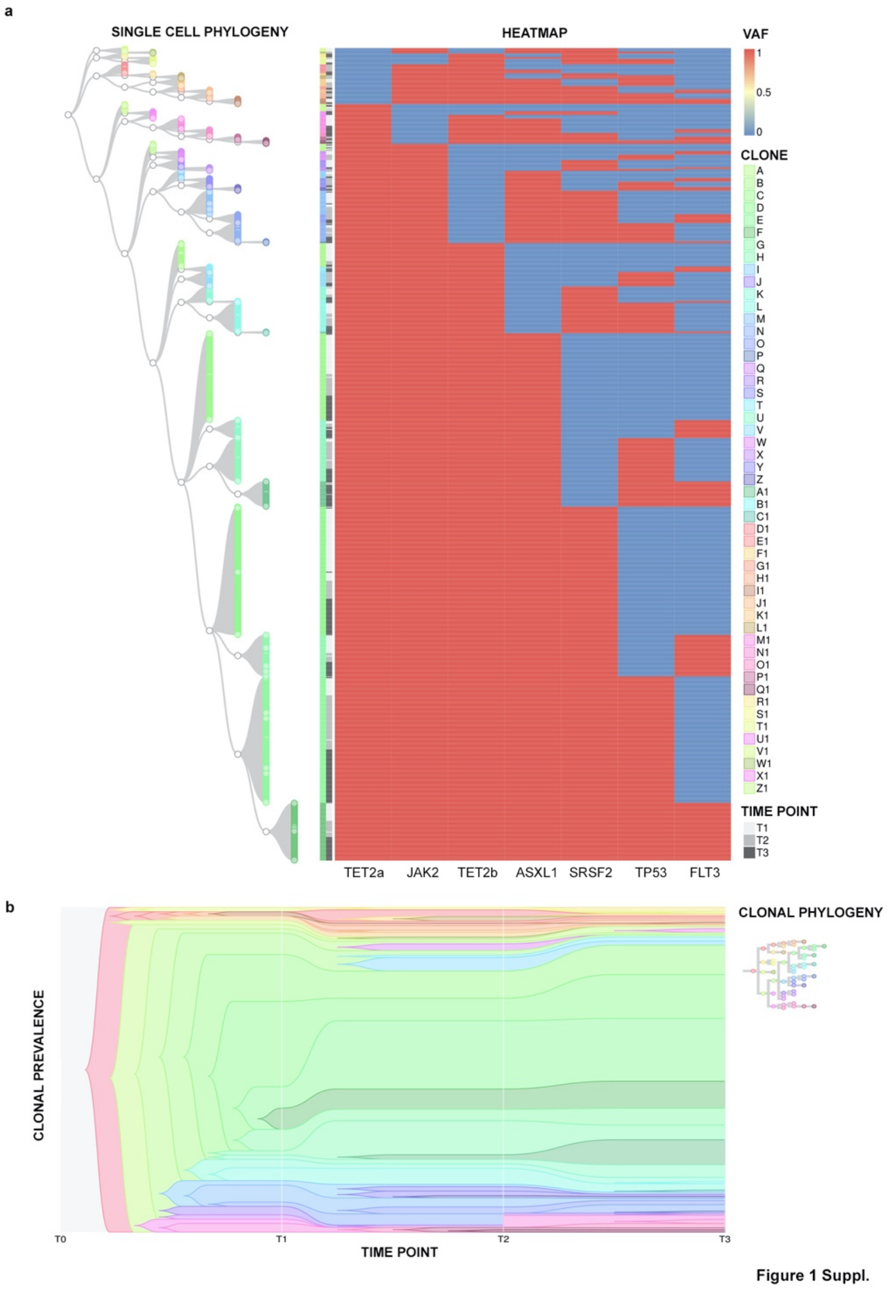

Supplementary Fig. 1. Single cell phylogeny and clone temporal evolution. Panel **a** shows the phylogenetic tree of 900 single cells analyzed in T1, T2 and T3. ***Left part:*** tree starting from a group of founder clones. Each clone (A-Z1) is represented by a color; nodes are represented as white circles. ***Right part:*** heatmap describing a mutational event (blue: wild-type, red: mutated) of the variants indicated in the bottom part of the graph. Each cell in the phylogenetic tree corresponds to a row in the heatmap, identifying its mutational profile. In Panel **b**, the Fishplot shows the abundance of the 52 clones (A-Z1), identified in Panel a, and their temporal evolution. Each clone is represented by the same color used in Panel a.

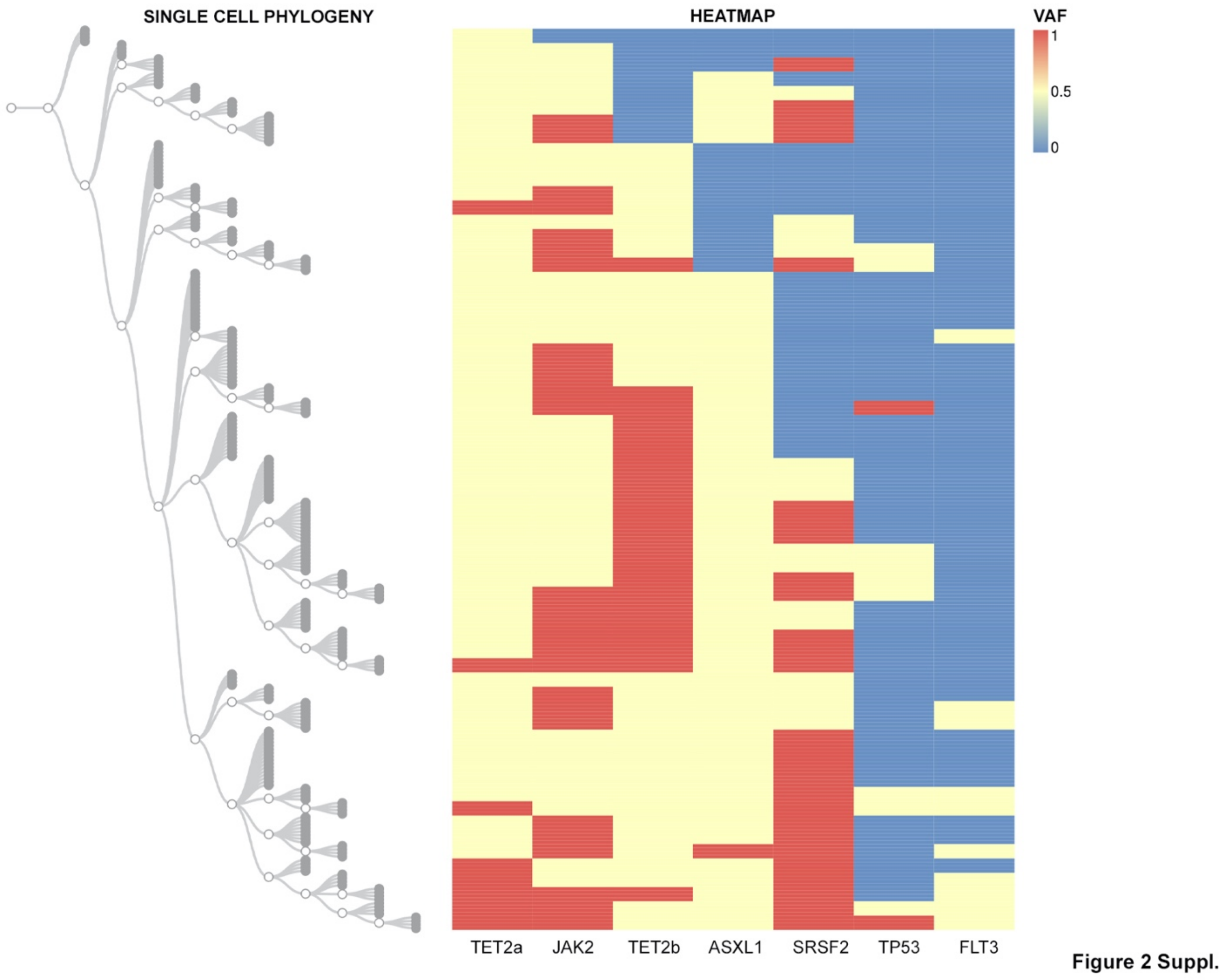

Supplementary Fig. 2 Single cell phylogeny of Time 1. Phylogenetic tree reconstruction built based on the zygosity of the different mutations in Time 1 and performed using CellScape R package. ***Left part:*** phylogenetic tree, black circles represents every single cell, the white circles represents the nodes of different clones. ***Right part:*** heatmap describing mutational event (blue: wild-type, yellow: heterozygous, red: homozygous) of the variants indicated in the bottom part of the graph. Each cell in the phylogenetic tree corresponds to a row in the heatmap, identifying its mutational profile.

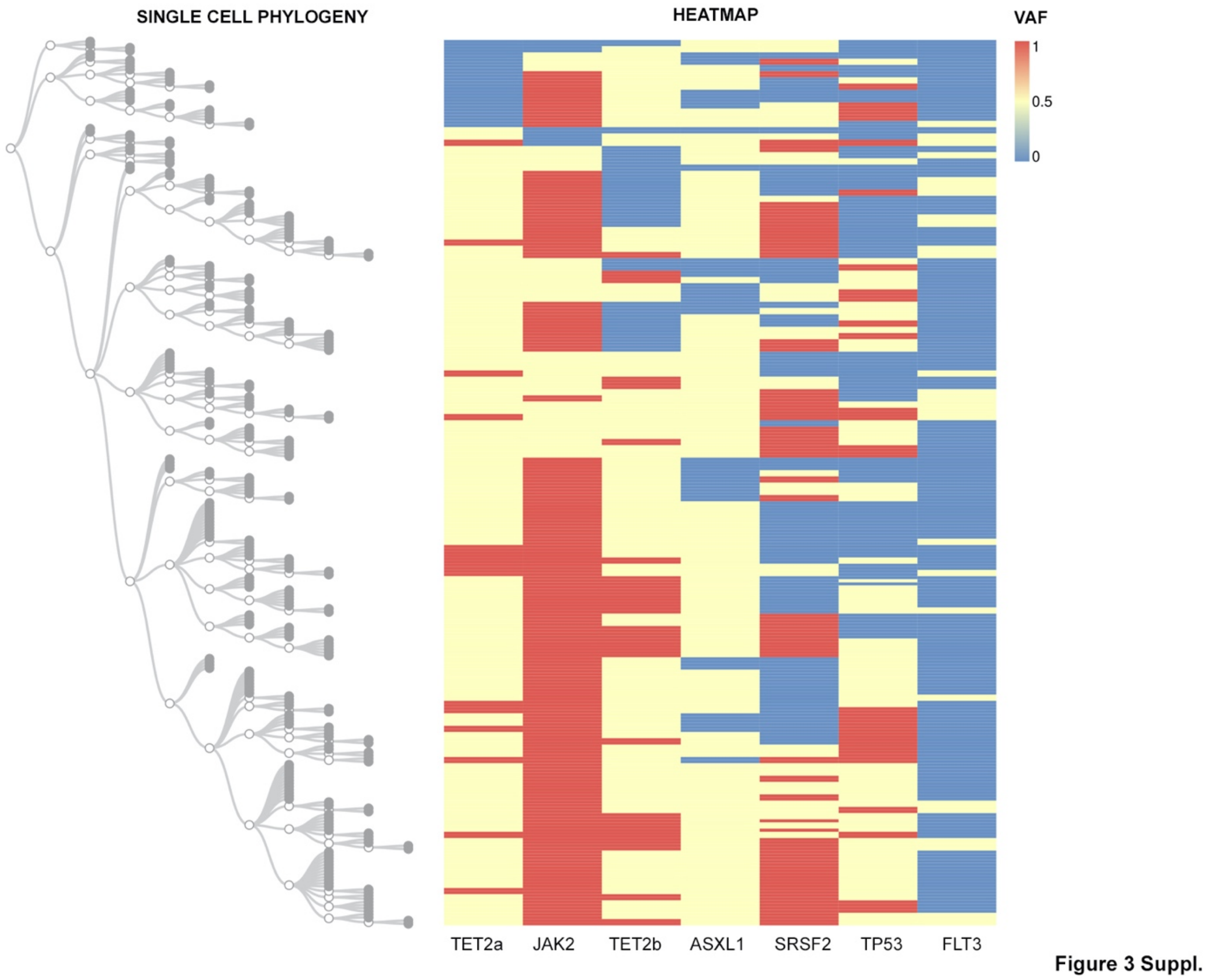

Supplementary Fig. 3 Single cell phylogeny of Time 2. Phylogenetic tree reconstruction built based on the zygosity of the different mutations in Time 2 and performed using CellScape R package. ***Left part:*** phylogenetic tree, black circles represents every single cell, the white circles represents the nodes of different clones. ***Right part:*** heatmap describing mutational event (blue: wild-type, yellow: heterozygous, red: homozygous) of the variants indicated in the bottom part of the graph. Each cell in the phylogenetic tree corresponds to a row in the heatmap, identifying its mutational profile.

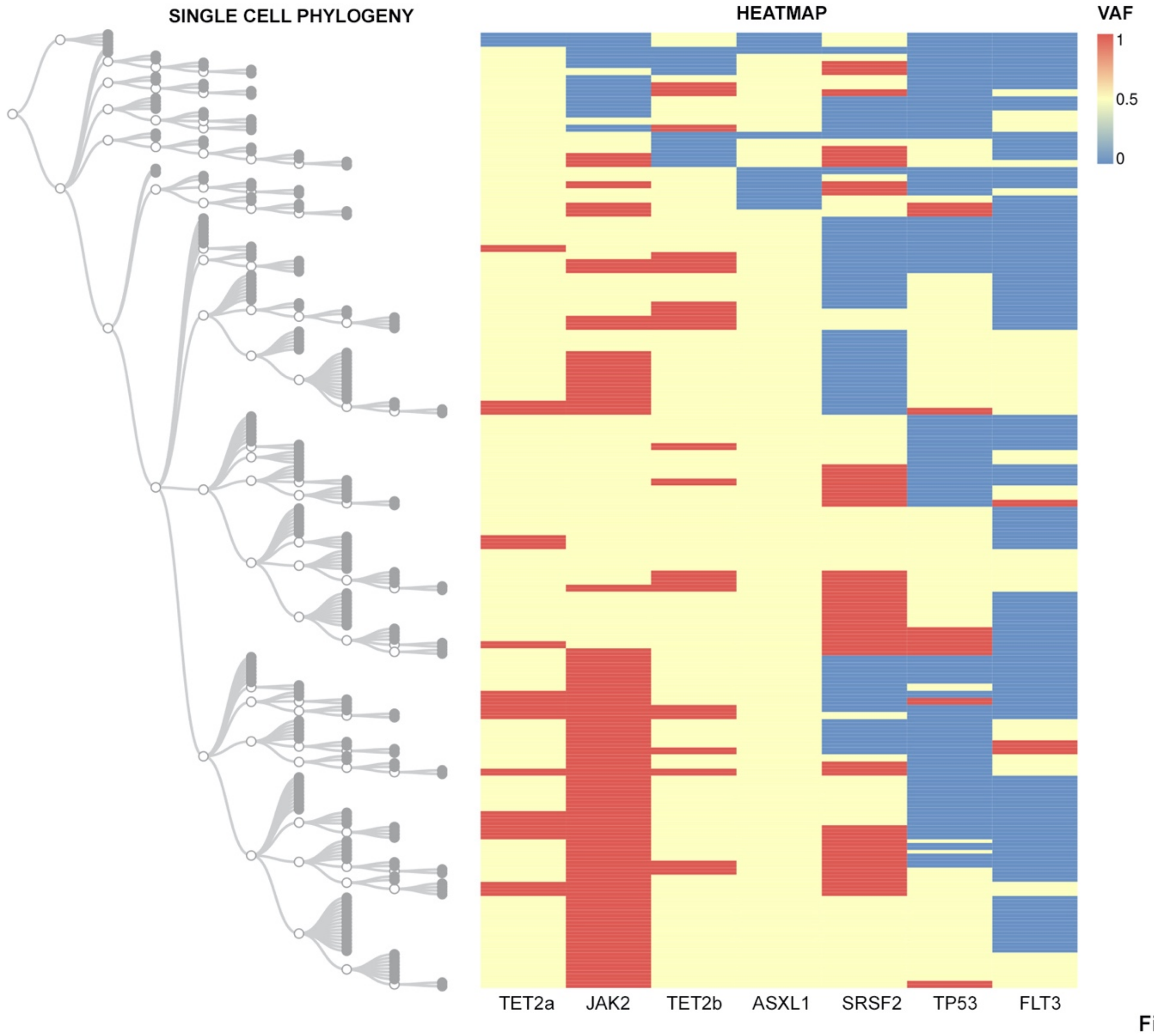

Figure 4 Suppl.

Supplementary Fig. 4 Single cell phylogeny of Time 3. Phylogenetic tree reconstruction built based on the zygosity of the different mutations in Time 3 and performed using CellScape R package. ***Left part:*** phylogenetic tree, black circles represents every single cell, the white circles represents the nodes of different clones. ***Right part:*** heatmap describing mutational event (blue: wild-type, yellow: heterozygous, red: homozygous) of the variants indicated in the bottom part of the graph. Each cell in the phylogenetic tree corresponds to a row in the heatmap, identifying its mutational profile.

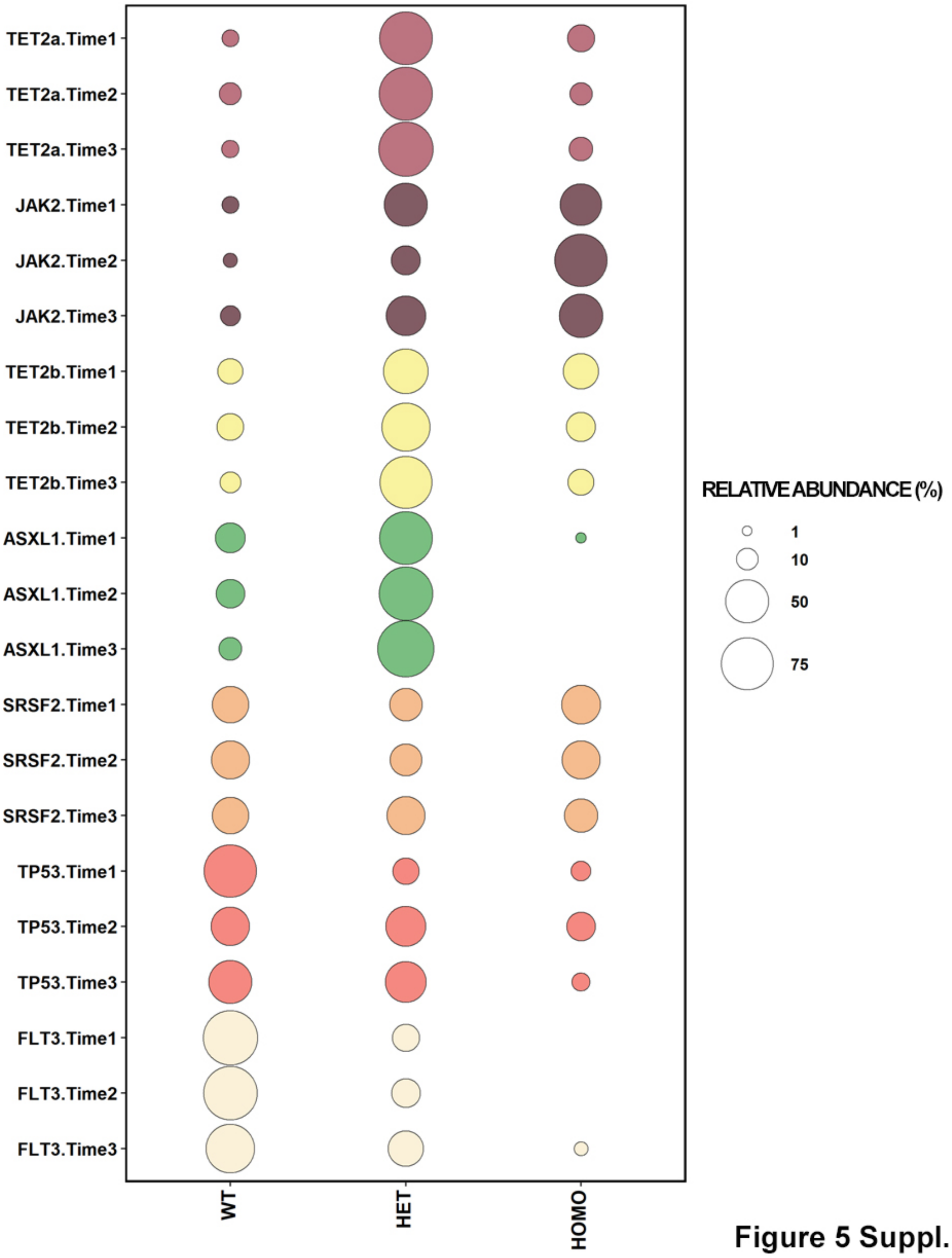

Supplementary Figure 5. **Zygosity distribution of all mutations in each time point.** Bubble plot of the frequencies of mutations through time performed by ggplot2 R package. On the y axis are represented all variants in Time 1, 2 and 3; on the x axis is shown the mutational status (WT: wild-type; HET: heterozygous; HOMO: homozygous). Each colour indicates a different variant. Circles' dimension indicates the relative abundance of the event.

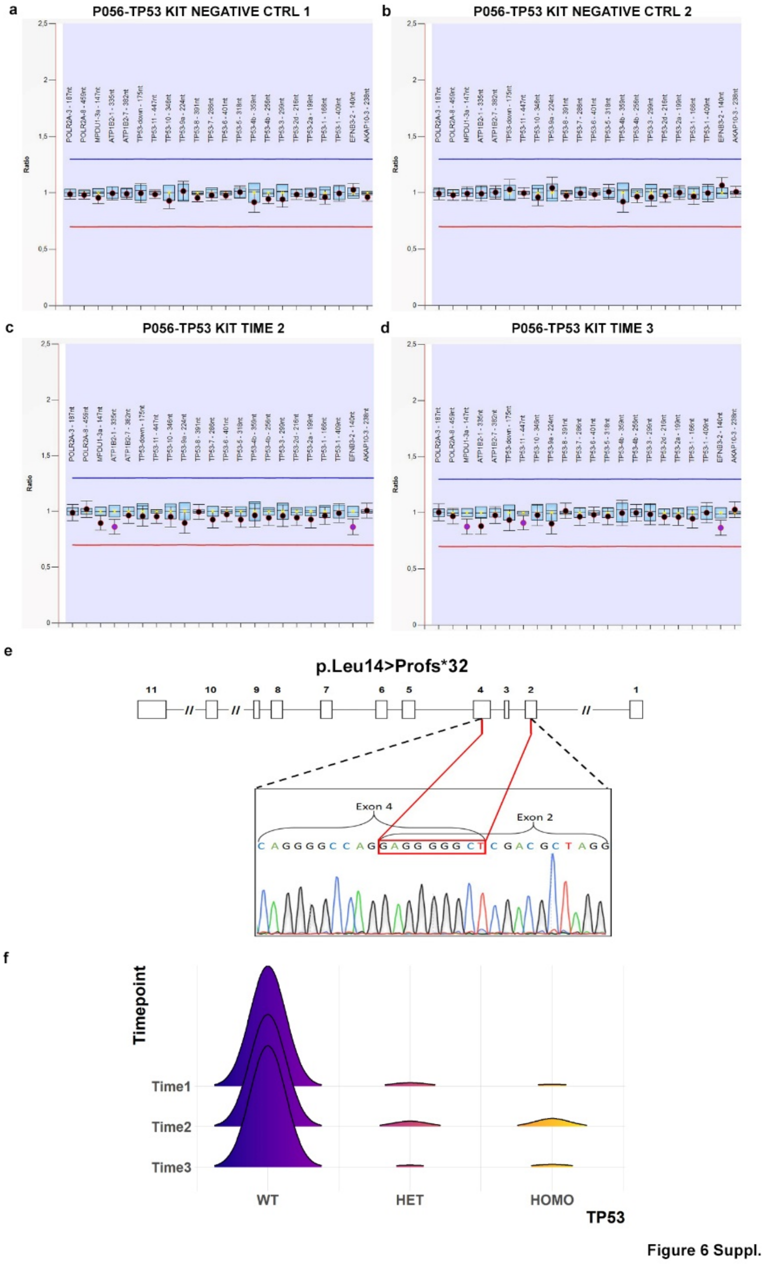

Supplementary Fig. 6. Characterization of *TP53* deletion. Panels **a-d** shows the results of Multiplex Ligation-dependent Probe Amplification (MLPA) analysis of ***TP53*** gene in bulk CD34+ cells. The data obtained from two healthy controls (Ctrl) (**a**, **b**), T2 (**c**) and T3 (**d**) do not show any CNVs. Panel **e** shows a deletion of 454bp in ***TP53*** gene (c.41_152del, p.Leu14>Profs*32) affecting a region between exons 2 and 4, detected by Sanger sequencing. In Panel **f** the Joy Plot shows the distribution of 454bp ***TP53*** deletion referred to all CD34+ cells analyzed in T1 (wt 96%, het 2,6%, homo 1,4%), T2 (wt 89,5%, het 4,2%, homo 6,3%) and T3 (wt 97,4%, het 0,65%, homo 1,95%).

Wt: wild-type; het: heterozygous; homo: homozygous.

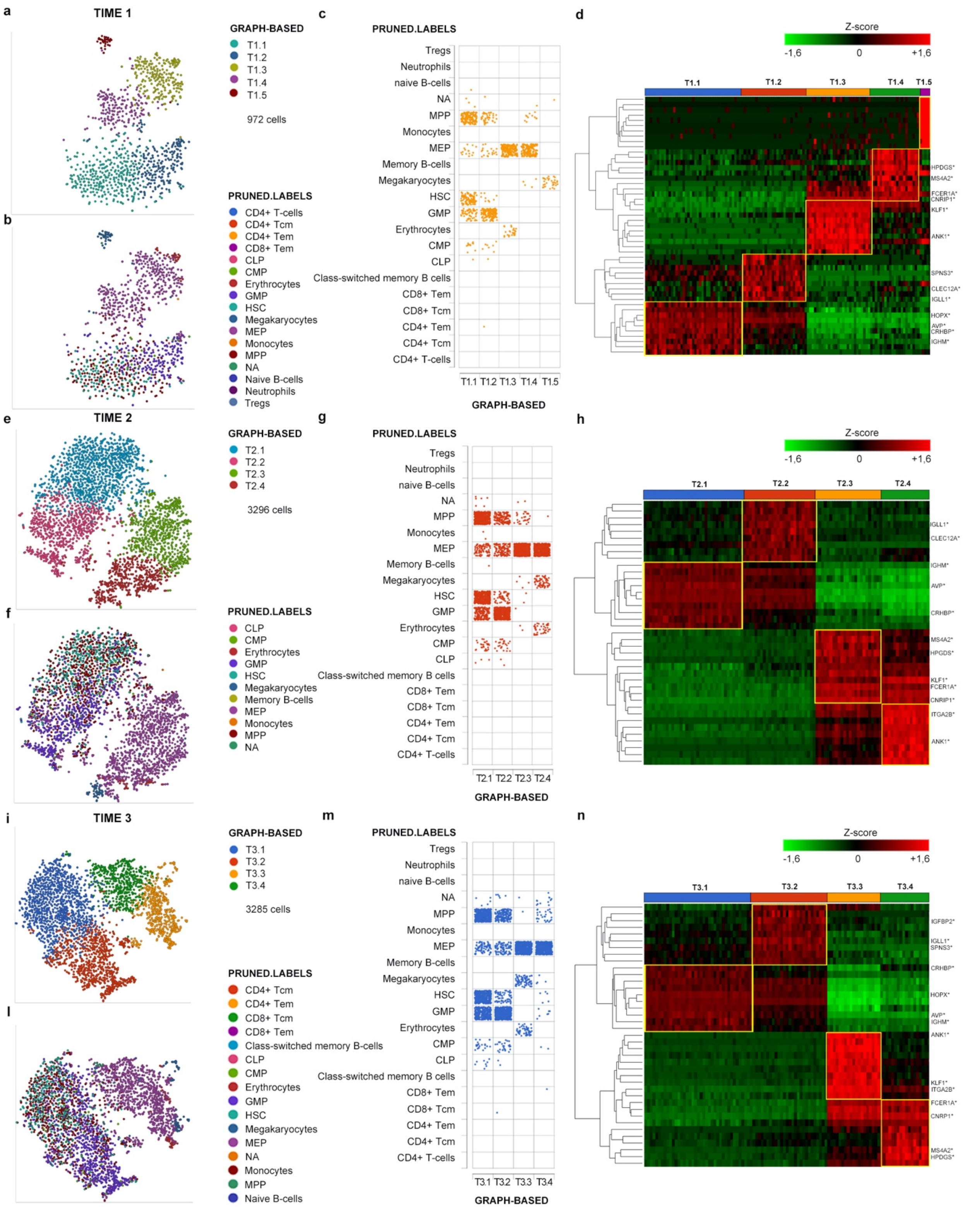

Figure 7 Suppl.

Supplementary figure 7: a tSNE projection representing clustering analysis result for T1 sample; in this sample 6 clusters were identified according to Partek Flow. Only 5 clusters are represented since one cluster was excluded due to the presence of contaminant cells. **b** Cells from T1 sample are colored in the tSNE projection according to the classification

obtained using SingleR package. The distribution of cells classified using SingleR in the 5 clusters identified in T1 is represented in panel **c. d**: The heatmap represents the supervised hierarchical clustering of cells in T1 and was obtained using the top 10 marker genes for each identified cluster. The same images were generated for T2 (**e** to **h**) and T3 (**i** to **n**) in order to allow the comparison between samples. In panels **d, h** and **n** marker genes shared by the three samples are shown.

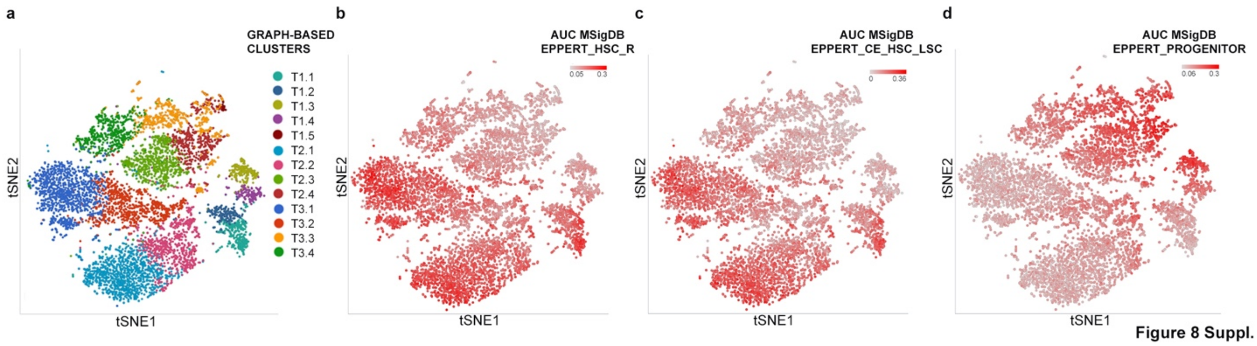

Supplementary Figure 8: tSNE projection of cells from all three samples. Data processing and analysis was performed by means of Partek® Flow® software; in these graphs 7553 cells were included. Panel **a** shows the different clusters identified by clustering analysis: 5 in T1 sample and 4 in both T2 and T3. In panels **b, c and d** cells are colored in red scale according to gene module activation, predicted using AUCell function in Partek® Flow® software. Gene signatures derived from Eppert et al. publication and retrieved from MSigDB was analyzed, in particular: **b** represents HSC related signature (EPPERT_HSC_R), **c** represents core enriched (CE) HSC-LSC genes (EPPERT_CE_HSC_LSC) (genes related to HSC features differentially expressed in LSC from AML samples) while panel **d** shows results relative to the progenitor cells' specific signature (EPPERT_PROGENITORS).

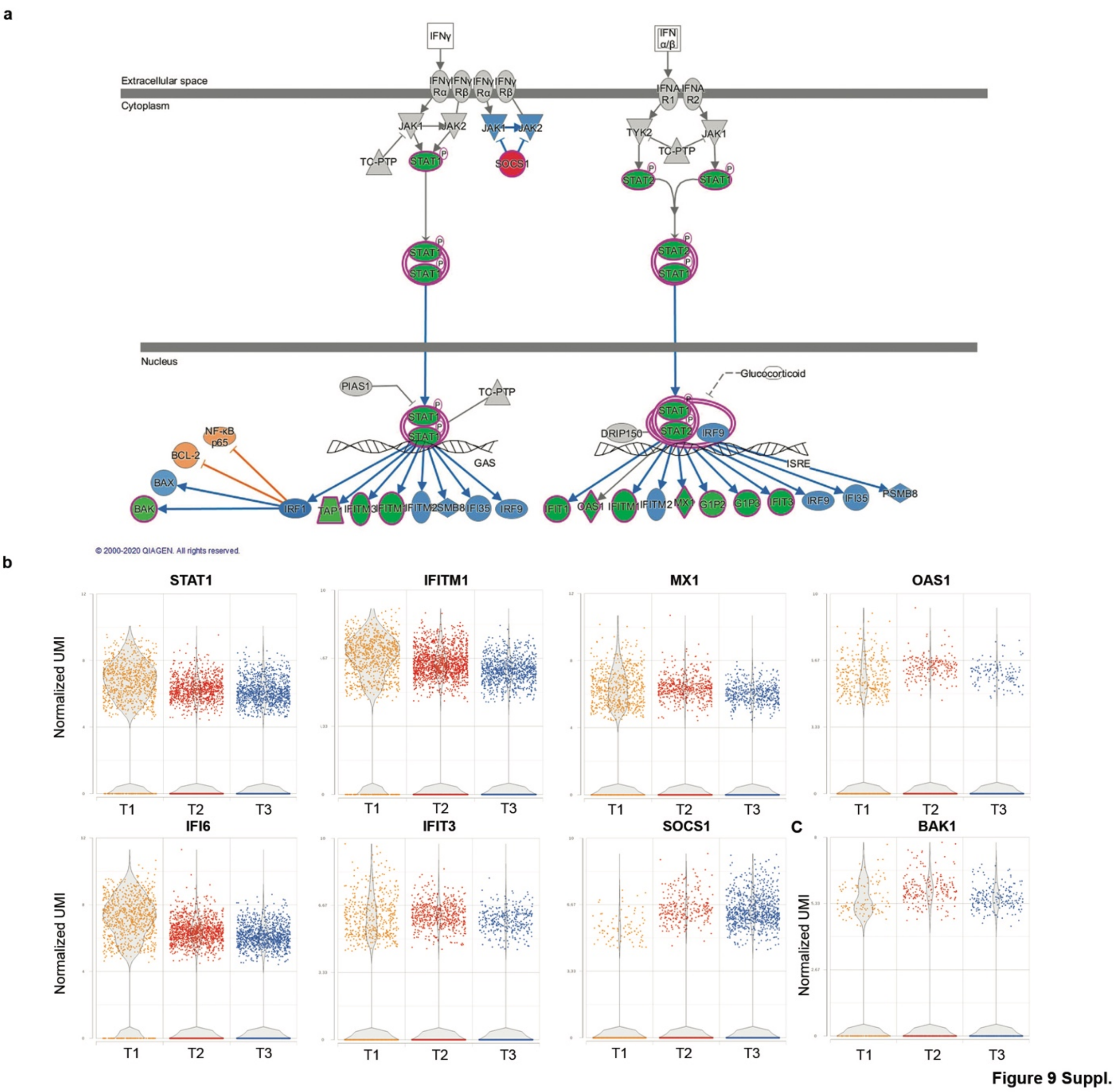

Supplementary Figure 9: Interferon signaling pathway. Panel **a** represents the canonical pathway "Interferon Signaling" as it is reported by IPA. Genes in green are downregulated while those in red are upregulated. Blue genes represent predicted inhibited upstream regulators, while orange genes are predicted activated ones. Figure shows differentially expressed genes in the comparison T3 vs T1 MPP_GMP cluster. Panel **b** includes dot plots generated from Partek® Flow® representing the expression of genes involved in IFN signaling according to IPA® in the three timepoints. Panel **c** shows the expression of BAK1 in cells belonging to MPP_GMP cluster in all the three samples.

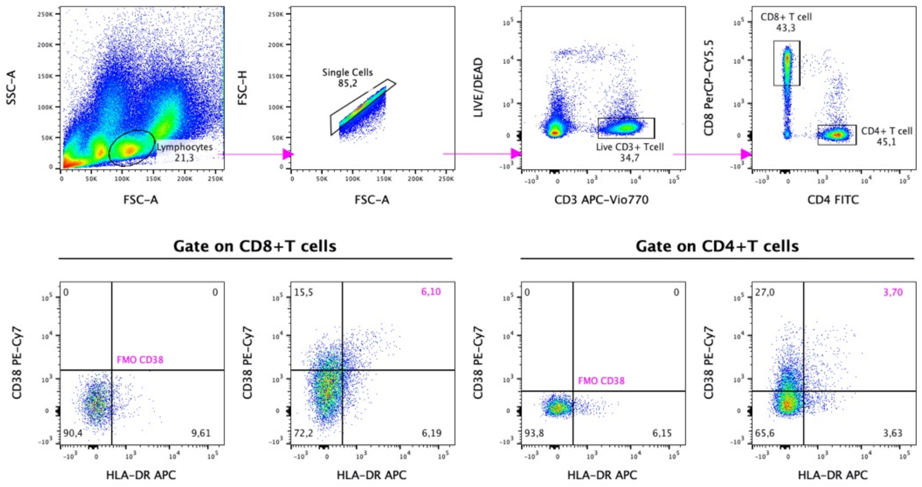

Figure 10 Suppl.

Supplementary Figure 10. Gating strategy for the detection of activated CD4+ and CD8+ T cells . Peripheral blood lymphocytes were gated based on SSC and FSC properties and doublets were excluded. Dead cells were excluded as negative to LIVE/DEAD dye. The expression of CD4 and CD8 were evaluated and the activation markers CD38 and HLA-DR were assessed within CD4+ and CD8+ T cells. FMO controls for CD38 and HLA-DR were used to sharply define the CD38+/HLA-DR+ population. The data refer to T2 and are representative of 10 independent experiments.

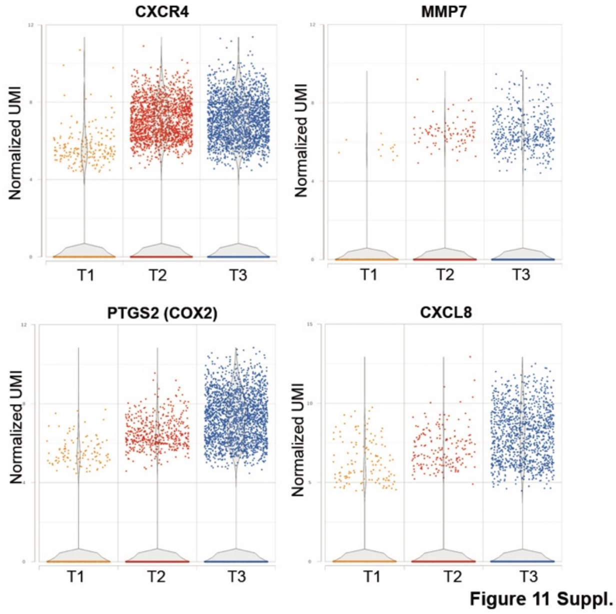

Supplementary Figure 11: Dot plots display the increased expression of genes related to extramedullary hematopoiesis in T3 sample compared to T1. Cells belonging to all the four clusters (HSC_MPP, MPP_GMP, MEP_1 and MEP_2) are included in this representation.

SUPPLEMENTARY TABLES

Supplementary Table 1: Primers used for PCR reactions in single cell sequencing

Gene	ID	Mutation (genomic coordinate hg38)	F	R
JAK2	Hs00532916_CE	g.5073770	TGAAGCAGCAAGTATGATGAG CAA	CTGACACCTAGCTGTGATC CTG
ASXL1	custom	g.32434646	CGGATCATCCCCACCACGGAG T	GGCGGCAGTAGTTGTGTTC GC
TET2	Hs00732331_CE	g.105243718	ATTGTGATTCTCATCCTGGTGT GG	AATGTAAAAGTGCACGCTG AACTC
TET2	Hs00629106_CE	g.105237351_105237 358	ATTGGATACACCTGTCAAGACT CAATATG	CAAGACACAAGCATCGGTA ACTTG
SRSF2	Hs00170120_CE	g.76736877	CGGCTGTGGTGTGAGTCCG	GCTGCCCGCCCAGTTGTTA
TP53	custom	g.7674250	AAGCCACAGGTTAAGAGGTCC C	AAAAGGCCTCCCCTGCTTG CC
FLT3	Hs00785316_CE	g.28018505	CCACAGTGAGTGCAGTTGTTTA C	AGAAGAAAGATTGCACTCC AGGATAATAC

Supplementary Table 2: % of TP53, FLT3 and SRSF2 mutated cells during disease progression

TP53	WT	HET	HOMO
Time1	76	16	8
Time2	38.02	42.25	19.71
Time3	49.34	44.07	6.57

FLT3	WT	HET	HOMO
Time1	82.6	17.3	0
Time2	80.4	19.5	0
Time3	64.9	31.8	3.2

SRSF2	WT	HET	HOMO
Time1	33.8	26.7	39.4
Time2	37.8	25	37.1
Time3	34.4	37.6	27.9

Supplementary Table 3: Frequency of cells classified according to SingleR using Blueprint ENCODE dataset in each cluster

	HSC_MPP		MPP_GMP		MEP_1		MEP_2	
SingleR Blueprint Classification	number	frequency	number	frequency	number	frequency	number	frequency
Tregs		0.00%		0.00%		0.00%		0.00%
Neutrophils		0.00%		0.00%		0.00%		0.00%
Naive B-cells	1	0.04%		0.00%		0.00%		0.00%
NA	14	0.52%	6	0.31%	15	1.00%	2	0.14%
MPP	1089	40.07%	332	17.26%	65	4.35%	2	0.14%
Monocytes	2	0.07%		0.00%		0.00%		0.00%
MEP	172	6.33%	238	12.38%	1381	92.50%	1119	78.86%
Memory B-Cells	1	0.04%		0.00%		0.00%		0.00%
Megakaryocytes		0.00%		0.00%	12	0.80%	179	12.61%
HSC	839	30.87%	119	6.19%	7	0.47%		0.00%
GMP	527	19.39%	1161	60.37%	8	0.54%		0.00%
Erythrocytes		0.00%		0.00%	2	0.13%	117	8.25%
CMP	61	2.24%	60	3.12%	2	0.13%		0.00%
CLP	12	0.44%	5	0.26%		0.00%		0.00%
Class-switched memory B-cells		0.00%		0.00%		0.00%		0.00%
CD8+ Tem		0.00%		0.00%	1	0.07%		0.00%
CD8+ Tcm		0.00%	1	0.05%		0.00%		0.00%
CD4+ Tem		0.00%	1	0.05%		0.00%		0.00%
CD4+ Tcm		0.00%		0.00%		0.00%		0.00%
CD4+ T-cells		0.00%		0.00%		0.00%		0.00%
Total	2718		1923		1493		1419	

Supplementary Table 4: number of differentially expressed genes (DEGs) in all comparisons

		DEGs |FC|>2 and FDR<0.05		
Comparison	Cluster	Tot	Up	Down
T3 vs T1	HSC_MPP	1091	340	751
T2 vs T1	HSC_MPP	576	268	308
T3 vs T1	MPP_GMP	854	266	588
T2 vs T1	MPP_GMP	472	226	246
T3 vs T1	MEP_1	1186	264	922
T2 vs T1	MEP_1	672	243	429
T3 vs T1	MEP_2	819	283	536
T2 vs T1	MEP_2	520	234	286

Supplementary Table 5: Lists of genes retrieved from literature and used for AUCell analysis in Partek Flow

VELTEN_FLT3_ SATB1_IND1	EPPERT_HSC_R		EPPERT_CE_ HSC_LSC	EPPERT_PROGENITOR	
TMSB4X	ABCB1	KDM2A	ABCB1	ACO1	MIPEP
ITM2C	ADGRG6	KLF4	ADGRG6	ACSM3	MRE11
FLT3	ALCAM	KMT2A	ALCAM	ADGRA3	MRPL35
LSP1	ANK3	KSR1	BAALC	AHCYL1	MSH3
SPNS3	ASXL1	LONP2	BCL11A	AKAP2	MTHFD2
PRAM1	ATP8B4	LPP	CACNB2	ANK1	MTMR2
CORO1A	BAALC	MAFK	CRHBP	ANKRD27	MTPAP
CAPG	BCL11A	MCTP1	DAPK1	ARMC8	MTREX
SEPT1	BEX1	MECOM	DRAM1	ASRGL1	MYCN
HLA-A	BTBD11	MEIS1	ELK3	BAMBI	NCBP1
HLA-B	C2orf69	MLLT3	ERG	BMS1	NDC1
SLC2A5	CACNB2	MREG	FAM30A	BTK	NDC80
VAMP5	CALN1	MSI2	FLT3	C5orf30	NET1
GSTP1	CD109	MSMO1	FRMD4B	CALU	NOMO1
SASH3	CFH	MYO5C	GUCY1A1	CCNB2	ODC1
SH3BGRL3	COL5A1	NPR3	HLA-DRB4	CCNJ	P2RY1
SORL1	CRHBP	OGT	HLF	CDK6	PAICS
ITGAL	CRIM1	OSER1	HOXA5	CDK7	PCLAF
SATB1	CYLD	PAM	HOXB2	CENPU	PCTP
HLA-DQB1	DACH1	PAN3-AS1	HOXB3	CHEK1	PDLIM1
LCP1	DAPK1	PCNX1	HTR1F	CHEK2	PDZD8
FNBP1	DDX5	PDE10A	INPP4B	CKAP5	PEX5
LAIR1	DLK1	PLSCR4	KAT6A	CNRIP1	PHF6
CD53	DNAJB9	PNISR	MECOM	CSNK2A1	PLIN2
GAPT	DRAM1	PNP	MEIS1	CTNNBL1	PLK4
SDK2	DST	PPP1R16B	MYO5C	CTPS1	PMP22
TPM4	DUSP6	PRKCH	PLSCR4	DCAF7	POLA1
ARPC2	EIF5	PROM1	PPP1R16B	DDAH1	POLE2
HLX	ELK3	PTK2	PRKCH	DLAT	PRKAR2B
RNASET2	EPC1	RBM7	RBPMS	DLC1	PRKDC
NEGR1	ERG	RBPMS	RNF125	DNAJC6	PSMD1
CD44	FAM106A	RCSD1	SLC25A36	DTL	RACGAP1
TNFSF8	FAM169A	RGCC	SMARCA1	DUT	RFC3
BCL11A	FAM30A	RIMKLB	SOCS2	EIF2AK1	RFX7
STK17B	FAM3C	RLIM	SPINK2	EIF2S1	RHAG
MAP1A	FGD5	RNF125	SPTBN1	EIF5B	RMDN1
LITAF	FLT3	RPL31	TCEAL9	EREG	RRM1
	FNBP1	RPS20P22	TFPI	FAM171A1	RYR3
	FOXO1	RSL1D1	TMEM38B	FANCI	SART3
	FRMD4B	RUNX2	YES1	FBXO7	SCD
	GCNT2	SEL1L3	ZNF165	FECH	SLC27A2
	GNL1	SLC17A9		FKBP14	SLF1
	GUCY1A1	SLC25A36		GINS2	SMC1A
	HES1	SMARCA1		HAUS6	SORD
	HIST1H2BD	SMARCA2		HBS1L	SPTA1
	HIST1H2BE	SOCS2		HDC	SSX2IP
	HIST1H2BG	SPINK2		HIRA	STAM
	HIST1H2BI	SPTBN1		HMGB1	STEAP3
	HIST2H2AA3	ST3GAL6		HMGXB4	SUPT16H
	HLA-DRB4	TCEAL9		HNRNPR	TAL1
	HLF	TCF12		HS2ST1	TFRC
	HOPX	TFPI		HSP90AA1	TIPIN
	HOXA5	TMEM107		IDE	TM9SF3
	HOXB2	TMEM200A		IDH3A	TMEM97
	HOXB3	TMEM38B		IPO5	TMOD3
	HTR1F	TPT1		KCNQ5	TNIK
	INPP4B	WDR91		KIFAP3	TPM1
	INSIG1	YES1		KNTC1	TPR
	IPO11	ZBTB4		LANCL2	TRIP13
	ITSN2	ZDHHC21		LDHB	TRIT1
	JUN	ZEB1		LHFPL2	TXNRD1
	KAT6A	ZNF165		LRPPRC	UBE2FP1
	KBTBD8			LRRFIP2	UMPS
				MARCH5	WASF1
				METAP2	WRN
				METTL14	XK
				MEX3B	ZNF225
				MICAL2	ZWINT
				MINPP1	

SUPPLEMENTARY DATA FILES LEGENDS

Supplementary Data File 1: The list of top-25 marker genes for each cluster in each sample ordered according to ascending p-value. It is also specified whether they are reported as marker genes for different hematopoietic stem/progenitor cells populations according to literature.

Supplementary Data File 2: The list of top-25 marker genes for each cell cluster identified by our classification ordered according to ascending p-value. It is also specified whether they are reported as marker genes for different hematopoietic stem/progenitor cells populations according to literature.

Supplementary Data File 3: The results of ANOVA analysis. For each identified cluster T3 and T2 sample was compared to T1. For each comparison Storey Q-value, false discovery rate step up p-value and fold change are reported. These data were used to perform core analysis by means of Ingenuity Pathway Analysis (IPA).

Supplementary Data File 4: The results of core analysis performed with Ingenuity Pathway Analysis (IPA). This sheet contains the list of canonical pathways predicted activated (z-score >2) or inhibited (z-score <2) in all comparisons.

Supplementary Data File 5: The results of core analysis performed with Ingenuity Pathway Analysis (IPA). This sheet contains the list of upstream regulators predicted activated (z-score >2) or inhibited (z-score <-2) in all comparisons.

Supplementary Data File 6: The results of core analysis performed with Ingenuity Pathway Analysis (IPA). This sheet contains the list of disease and function categories predicted activated (z-score >2) or inhibited (z-score <-2) in all comparisons.

REFERENCES

1 Eppert, K. *et al.* Stem cell gene expression programs influence clinical outcome in human leukemia. *Nat Med* **17**, 1086-1093, doi:10.1038/nm.2415 (2011).

2 Velten, L. *et al.* Human haematopoietic stem cell lineage commitment is a continuous process. *Nat Cell Biol* **19**, 271-281, doi:10.1038/ncb3493 (2017).

3 Hay, S. B., Ferchen, K., Chetal, K., Grimes, H. L. & Salomonis, N. The Human Cell Atlas bone marrow single-cell interactive web portal. *Exp Hematol* **68**, 51-61, doi:10.1016/j.exphem.2018.09.004 (2018).

4 Zheng, S., Papalexi, E., Butler, A., Stephenson, W. & Satija, R. Molecular transitions in early progenitors during human cord blood hematopoiesis. *Mol Syst Biol* **14**, e8041, doi:10.15252/msb.20178041 (2018).

5 Palpant, N. J. *et al.* Chromatin and Transcriptional Analysis of Mesoderm Progenitor Cells Identifies HOPX as a Regulator of Primitive Hematopoiesis. *Cell Rep* **20**, 1597-1608, doi:10.1016/j.celrep.2017.07.067 (2017).

6 He, X. *et al.* Differential gene expression profiling of CD34+ CD133+ umbilical cord blood hematopoietic stem progenitor cells. *Stem Cells Dev* **14**, 188-198, doi:10.1089/scd.2005.14.188 (2005).

7 Sommerkamp, P. *et al.* The long non-coding RNA Meg3 is dispensable for hematopoietic stem cells. *Sci Rep* **9**, 2110, doi:10.1038/s41598-019-38605-8 (2019).

8 Liu, J. *et al.* Immunoglobulin gene expression in umbilical cord blood-derived CD34(+) hematopoietic stem/progenitor cells. *Gene* **575**, 108-117, doi:10.1016/j.gene.2015.08.046 (2016).

9 Bill, M. *et al.* Mapping the CLEC12A expression on myeloid progenitors in normal bone marrow; implications for understanding CLEC12A-related cancer stem cell biology. *J Cell Mol Med* **22**, 2311-2318, doi:10.1111/jcmm.13519 (2018).

10 Inage, E. *et al.* Critical Roles for PU.1, GATA1, and GATA2 in the expression of human FcepsilonRI on mast cells: PU.1 and GATA1 transactivate FCER1A, and GATA2 transactivates FCER1A and MS4A2. *J Immunol* **192**, 3936-3946, doi:10.4049/jimmunol.1302366 (2014).

11 Psaila, B. *et al.* Single-cell profiling of human megakaryocyte-erythroid progenitors identifies distinct megakaryocyte and erythroid differentiation pathways. *Genome Biol* **17**, 83, doi:10.1186/s13059-016-0939-7 (2016).

12 Li, Y., Qi, X., Liu, B. & Huang, H. The STAT5-GATA2 pathway is critical in basophil and mast cell differentiation and maintenance. *J Immunol* **194**, 4328-4338, doi:10.4049/jimmunol.1500018 (2015).

13 Bianchi, E. *et al.* MYB controls erythroid versus megakaryocyte lineage fate decision through the miR-486-3p-mediated downregulation of MAF. *Cell Death Differ* **22**, 1906-1921, doi:10.1038/cdd.2015.30 (2015).

14 Satchwell, T. J. *et al.* Severe Ankyrin-R deficiency results in impaired surface retention and lysosomal degradation of RhAG in human erythroblasts. *Haematologica* **101**, 1018-1027, doi:10.3324/haematol.2016.146209 (2016).

15 Gorgens, A. *et al.* Revision of the human hematopoietic tree: granulocyte subtypes derive from distinct hematopoietic lineages. *Cell Rep* **3**, 1539-1552, doi:10.1016/j.celrep.2013.04.025 (2013).

16 Elli, E. M., Barate, C., Mendicino, F., Palandri, F. & Palumbo, G. A. Mechanisms Underlying the Anti-inflammatory and Immunosuppressive Activity of Ruxolitinib. *Front Oncol* **9**, 1186, doi:10.3389/fonc.2019.01186 (2019).

17 Bottos, A. *et al.* Decreased NK-cell tumour immunosurveillance consequent to JAK inhibition enhances metastasis in breast cancer models. *Nat Commun* **7**, 12258, doi:10.1038/ncomms12258 (2016).
18 Keskinen, P., Ronni, T., Matikainen, S., Lehtonen, A. & Julkunen, I. Regulation of HLA class I and II expression by interferons and influenza A virus in human peripheral blood mononuclear cells. *Immunology* **91**, 421-429, doi:10.1046/j.1365-2567.1997.00258.x (1997).
19 Rodriguez, J. A. HLA-mediated tumor escape mechanisms that may impair immunotherapy clinical outcomes via T-cell activation. *Oncol Lett* **14**, 4415-4427, doi:10.3892/ol.2017.6784 (2017).
20 Lemoli, R. M. *et al.* Molecular and functional analysis of the stem cell compartment of chronic myelogenous leukemia reveals the presence of a CD34- cell population with intrinsic resistance to imatinib. *Blood* **114**, 5191-5200, doi:10.1182/blood-2008-08-176016 (2009).
21 Matatall, K. A., Shen, C. C., Challen, G. A. & King, K. Y. Type II interferon promotes differentiation of myeloid-biased hematopoietic stem cells. *Stem Cells* **32**, 3023-3030, doi:10.1002/stem.1799 (2014).
22 Fatehchand, K. *et al.* Interferon-gamma Promotes Antibody-mediated Fratricide of Acute Myeloid Leukemia Cells. *J Biol Chem* **291**, 25656-25666, doi:10.1074/jbc.M116.753145 (2016).
23 Croker, B. A. *et al.* Fas-mediated neutrophil apoptosis is accelerated by Bid, Bak, and Bax and inhibited by Bcl-2 and Mcl-1. *Proc Natl Acad Sci U S A* **108**, 13135-13140, doi:10.1073/pnas.1110358108 (2011).
24 Austin, R. J. *et al.* Distinct effects of ruxolitinib and interferon-alpha on murine JAK2V617F myeloproliferative neoplasm hematopoietic stem cell populations. *Leukemia* **34**, 1075-1089, doi:10.1038/s41375-019-0638-y (2020).
25 Lin, S. *et al.* A FOXO1-induced oncogenic network defines the AML1-ETO preleukemic program. *Blood* **130**, 1213-1222, doi:10.1182/blood-2016-11-750976 (2017).
26 Firat, E. & Niedermann, G. FoxO proteins or loss of functional p53 maintain stemness of glioblastoma stem cells and survival after ionizing radiation plus PI3K/mTOR inhibition. *Oncotarget* **7**, 54883-54896, doi:10.18632/oncotarget.10702 (2016).
27 Min, I. M. *et al.* The transcription factor EGR1 controls both the proliferation and localization of hematopoietic stem cells. *Cell Stem Cell* **2**, 380-391, doi:10.1016/j.stem.2008.01.015 (2008).
28 Cheng, H. *et al.* Leukemic marrow infiltration reveals a novel role for Egr3 as a potent inhibitor of normal hematopoietic stem cell proliferation. *Blood* **126**, 1302-1313, doi:10.1182/blood-2015-01-623645 (2015).
29 Sirin, O., Lukov, G. L., Mao, R., Conneely, O. M. & Goodell, M. A. The orphan nuclear receptor Nurr1 restricts the proliferation of haematopoietic stem cells. *Nat Cell Biol* **12**, 1213-1219, doi:10.1038/ncb2125 (2010).
30 Cheng, H. *et al.* Novel regulators in hematopoietic stem cells can be revealed by a functional approach under leukemic condition. *Leukemia* **30**, 2074-2077, doi:10.1038/leu.2016.118 (2016).
31 Tian, C. *et al.* Hes1 mediates the different responses of hematopoietic stem and progenitor cells to T cell leukemic environment. *Cell Cycle* **12**, 322-331, doi:10.4161/cc.23160 (2013).
32 Roberts, A. S. *et al.* Extramedullary haematopoiesis: radiological imaging features. *Clin Radiol* **71**, 807-814, doi:10.1016/j.crad.2016.05.014 (2016).
33 Cheng, H., Sun, G. & Cheng, T. Hematopoiesis and microenvironment in hematological malignancies. *Cell Regen (Lond)* **7**, 22-26, doi:10.1016/j.cr.2018.08.002 (2018).

34 Song, M. K., Park, B. B. & Uhm, J. E. Understanding Splenomegaly in Myelofibrosis: Association with Molecular Pathogenesis. *Int J Mol Sci* **19**, doi:10.3390/ijms19030898 (2018).

35 Rosti, V. *et al.* The expression of CXCR4 is down-regulated on the CD34+ cells of patients with myelofibrosis with myeloid metaplasia. *Blood Cells Mol Dis* **38**, 280-286, doi:10.1016/j.bcmd.2007.01.003 (2007).

36 Spoo, A. C., Lubbert, M., Wierda, W. G. & Burger, J. A. CXCR4 is a prognostic marker in acute myelogenous leukemia. *Blood* **109**, 786-791, doi:10.1182/blood-2006-05-024844 (2007).

37 Lynch, C. C. & McDonnell, S. The role of matrilysin (MMP-7) in leukaemia cell invasion. *Clin Exp Metastasis* **18**, 401-406, doi:10.1023/a:1010973808853 (2000).

38 Zhang, J. *et al.* MMP-7 is upregulated by COX-2 and promotes proliferation and invasion of lung adenocarcinoma cells. *Eur J Histochem* **58**, 2262, doi:10.4081/ejh.2014.2262 (2014).

39 Obermajer, N., Muthuswamy, R., Odunsi, K., Edwards, R. P. & Kalinski, P. PGE(2)-induced CXCL12 production and CXCR4 expression controls the accumulation of human MDSCs in ovarian cancer environment. *Cancer Res* **71**, 7463-7470, doi:10.1158/0008-5472.CAN-11-2449 (2011).

40 Pold, M. *et al.* Cyclooxygenase-2-dependent expression of angiogenic CXC chemokines ENA-78/CXC Ligand (CXCL) 5 and interleukin-8/CXCL8 in human non-small cell lung cancer. *Cancer Res* **64**, 1853-1860, doi:10.1158/0008-5472.can-03-3262 (2004).

41 Stamatakis, K. *et al.* Prostaglandins induce early growth response 1 transcription factor mediated microsomal prostaglandin E2 synthase up-regulation for colorectal cancer progression. *Oncotarget* **6**, 39941-39959, doi:10.18632/oncotarget.5402 (2015).

42 Yakar, I. *et al.* Prostaglandin e(2) suppresses NK activity in vivo and promotes postoperative tumor metastasis in rats. *Ann Surg Oncol* **10**, 469-479, doi:10.1245/aso.2003.08.017 (2003).

43 Gong, X., Didan, Y., Lock, J. G. & Stromblad, S. KIF13A-regulated RhoB plasma membrane localization governs membrane blebbing and blebby amoeboid cell migration. *EMBO J* **37**, doi:10.15252/embj.201898994 (2018).
